# Macrolipasemia secondary to colon cancer chemotherapy: a case report

**DOI:** 10.11613/BM.2021.030801

**Published:** 2021-10-15

**Authors:** Hatice Saracoglu, Gulden Baskol, Mevlut Baskol

**Affiliations:** 1 Department of Medical Biochemistry, Medical Faculty, Erciyes University, Kayseri, Turkey; 2 Department of Gastroenterology, Medical Faculty, Erciyes University, Kayseri, Turkey

**Keywords:** macrolipasemia, chemotherapy, oxaliplatin, capecitabine, pancreatitis

## Abstract

We reported macrolipasemia in a colon cancer patient during the chemotherapy period without any evidence of pancreatitis. A 52-year-old man formerly treated for papillary thyroid carcinoma had elevated a carcinoembryonic antigen (CEA) concentration in the latest control and was diagnosed with colon cancer. Xelox chemotherapy (oxaliplatin and capecitabine) protocol was planned for six months. Interestingly, the lipase activities gradually increased from 30 U/L to 434 U/L, and exceeded three times the upper limit of the reference range (13-60 U/L). There were no symptoms of pancreatitis, and the abdominal computed tomography (CT) scan was also normal. Polyethylene glycol (PEG) recovery % values of serum samples gradually decreased and were 27% in the recent sample before the end of chemotherapy. Interestingly, the serum lipase activity fell a month after chemotherapy, and PEG recovery % increased (39%). We considered the following possibilities: (1) macrolipasemia due to chemotherapy drugs, (2) macrolipasemia due to antibodies against chemotherapy drugs.

## Introduction

Elevated serum amylase and lipase activities are important in the diagnosis of pancreatitis. A high amylase is not specific to pancreas disease and may be associated with other clinical conditions. Increased lipase activity associated with patients’ clinical symptoms is a more reliable and specific test for pancreatic disease (*1*, *2*). However, lipase elevation due to macrolipasemia is an extremely rare condition, such that there have been very few reports of macrolipasemia in the literature.

Macroenzymes are high molecular weight forms of plasma enzymes occurred by self-polymerization or binding to other plasma components, cannot be excreted by the kidneys, and cause increased enzyme activities. They are divided into two groups. It is known that immunoglobulins form complexes with various plasma enzymes. Immunoglobulin complexed enzymes are Type 1 macroenzymes. Type 2 macroenzymes are enzymes complexed with non-immunoglobulin plasma components (for example, lipoproteins, xenobiotics) or made by self-polymerization. One of these components may also be foreign substances (for example, drugs) (*3*, *4*).

Macroenzymes are suspected in atypical clinical cases associated with high serum enzyme concentrations (*5*). Macroenzymes must be detected as they may cause false high serum enzyme results and, therefore, diagnostic and therapeutic errors (*4*).

Unlike the literature, we reported macrolipasemia in a colon cancer patient during the chemotherapy period without any evidence of pancreatitis.

## Case

**Figure 1:**
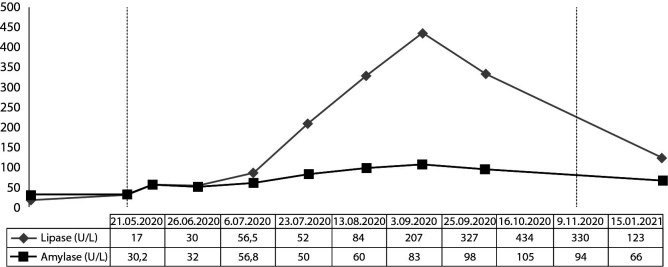
Changes in serum amylase and lipase activities in a patient. The patient received his first treatment on June 26, 2020 (left vertical line) and the last on December 15, 2020 (right vertical line). After the first treatment, lipase values increased rapidly. The accompanying amylase values remained within the reference range.

In the latest control, a 52-year-old man formerly treated for papillary thyroid carcinoma had elevated a carcinoembryonic antigen (CEA) concentration. Measured CEA value was 12 µg/L (reference range: 0-6.5 µg/L). Furthermore, a lesion was detected by colonoscopy. The patient was operated on and diagnosed with Stage 3 (T3/N1/M0) colon cancer. Xelox chemotherapy (oxaliplatin and capecitabine) protocol was planned for six months. Routine biochemical tests were followed during chemotherapy. Interestingly, the lipase activities gradually increased and exceeded three times the upper limit of the reference range (13-60 U/L). Almost all simultaneously measured amylase activities were in the reference range (28-100 U/L) (Figure 1). The patient had not previously been on drugs other than 150 µg of levotiron, cigarettes, and alcohol. There were no symptoms of pancreatitis (abdominal pain, nausea, vomiting, and fever), and the abdominal computed tomography (CT) scan was also normal. We suspected macrolipasemia in the patient without clinical and radiological signs of pancreatitis. The patient signed an informed consent form for anonymous publication of medical data.

## Methods

The blood samples taken into serum separator tubes (Vacuette, Greiner Bio-One GmbH, Kremsmünster, Austria) were centrifuged at 2000xg for 10 minutes, and serum samples were separated. Fresh serum samples’ amylase and lipase activities were performed on Cobas c701 (Roche Diagnostics, Mannheim, Germany) with original Roche test kits and enzymatic colorimetric methods. The lipase activity in the serum sample taken on September 25, 2020 was additionally evaluated on Architect c16000 (Abbott Diagnostics, Massachusetts, United States) with original Abbott test kits and enzymatic colorimetric methods to exclude analytical errors.

Lipase activities were measured after polyethylene glycol (PEG) (PEG 6000, CAS-No: 25322-68-3, Merck KGaA, Darmstadt, Germany) precipitation to evaluate macrolipasemia. After mixing 200 µL of serum and 200 µL of 25% PEG, mixture was incubated at room temperature for 10 minutes. Then it was centrifuged at 13000xg for five minutes. Lipase activity was determined in the supernatant, and PEG recovery % was calculated according to the following formula:

PEG recovery % = (Post-PEG lipase activity / Pre-PEG lipase activity) x 100.

In addition, Immunoglobulin (Ig)G, IgA, IgM, total kappa, and total lambda concentrations in the blood sample taken on September 25, 2020 were evaluated on Cobas e801 (Roche Diagnostics, Mannheim, Germany) with original Roche test kits and electrochemiluminescence immunoassay (ECLIA) methods.

## Results

PEG recovery % values of serum samples gradually decreased and were 27% in the recent sample before the end of chemotherapy. However, a month after chemotherapy, the serum lipase activity decreased (123 U/L), and PEG recovery % increased (39%) (Table 1).

**Table 1:** Changes in PEG recovery % of lipase

	**25.09.2020**	**16.10.2020**	**09.11.2020**	**15.01.2021**
Pre-PEG lipase (U/L)	327	434	330	123
Post-PEG lipase (U/L)	122	128	90	48
PEG recovery	37%	29%	27%	39%
Lipase PEG recovery % values of the patient gradually decreased during chemotherapy and became compatible with macrolipasemia (< 32%). A month after the end of chemotherapy (last column), it increased dramatically. PEG - polyethylene glycol.				

The lipase activities in the same serum sample (September 25, 2020) were measured as 327 U/L on Roche and 238 U/L on Abbott, and consequently, the analytical error was excluded.

Serum IgG, A, M, total kappa, and lambda concentrations are shown in Table 2. The increased total kappa concentration is striking.

**Table 2:** Immunoglobulin (Ig)G, IgA, IgM, total kappa and total lambda concentrations on September 25, 2020.

**Test**	**Result**	**Reference Range**
IgG	14.83 g/L	7-16
IgA	2.34 g/L	0.7-4.0
IgM	0.907 g/L	0.4-2.3
Total kappa	4.24 g/L ▲	1.56-4.08
Total lambda	2.01 g/L	0.83-2.24

## Discussion

Initially in this case, high lipase activities brought to mind acute pancreatitis, but clinical and radiological evidence was not present. The persistence of lipase elevation, absence of concomitant amylase elevation, and clinical incompatibility with pancreatitis suggested macrolipasemia.

There were no inconsistencies in the current autoanalyser’s internal and external quality control data (Roche), and we also detected high lipase activity on an alternative autoanalyser (Abbott). Therefore, we excluded the analytical error.

The PEG recovery % reference range for lipase is reported as 32–75%. Recovery % values lower than 32% are in favour of macrolipasemia (*6*). In this case, we found that lipase PEG recovery % values were 29% and 27% (< 32%) during the chemotherapy process. These findings supported that the elevated lipase activity in the patient were due to macrolipasemia.

Previously, macrolipasemia has been reported in autoimmune diseases, inflammatory diseases, and lymphoma (*7*-*10*). For the first time, we reported macrolipasemia secondary to colon cancer chemotherapy.

Different tumour types have been shown to express lipase, and there are reported cases of tumor-derived lipase-related lipase elevation (*11*-*13*). It has been shown that lipase activities rapidly decrease after treatment (*13*). In this case, on the contrary, lipase activities increased after chemotherapy. Therefore, we did not consider tumor-derived lipase-related lipase elevation.

In the literature, there are previously reported few cases of oxaliplatin and capecitabine-induced pancreatitis. Clinical, laboratory and radiological evidence of acute pancreatitis is available in all those cases (*14*, *15*).

It is known that enzymes can transform to the macroenzyme by forming a drug complex (*3*). Since lipase activities gradually increase during chemotherapy and decrease after the end of chemotherapy (PEG recovery % values are the opposite of these), we considered the following possibilities in this patient: (*1*) macrolipasemia due to lipase complexation with one or both of the chemotherapy drugs (oxaliplatin and capecitabine); (*2*) macrolipasemia due to lipase complexation with advanced antibodies against one or both chemotherapy drugs. The elimination half-life of oxaliplatin is 12-50 days, and the elimination half-life of capecitabine is approximately 30-50 minutes (*16*, *17*). The return of macrolipasemia a month after chemotherapy suggests that it may be mainly related to oxaliplatin.

Besides, the high value of total kappa suggests antibody-related macrolipasemia. There are reported cases of macroenzymes complexed with kappa in the literature (*18*).

During chemotherapy, the development of pancreatitis is an indication to stop treatment. Therefore, it is essential to reveal the presence of macrolipasemia in this group of patients.

In conclusion, in patients receiving chemotherapy, it should be kept in mind that drugs may trigger macroenzyme formation (drug and/or antibodies against drug) and cause enzyme elevations incompatible with the patients’ clinical condition.
